# Performance evaluation of a low-cost real time COVID-19 health monitoring system

**DOI:** 10.1186/s43067-023-00092-3

**Published:** 2023-06-14

**Authors:** Mayada Abdelgadir Ahmed, Monzir Hashim Mohammed, Ibrahim Hassan Ahmed

**Affiliations:** Electronics Engineering School, College of Engineering, Sudan University of Science and Technology (SUST), Khartoum, Sudan

**Keywords:** COVID-19, Health monitoring, Sensor network, Performance evaluation, Remote health model

## Abstract

Recently, and to cater for increased needs on health monitoring and data management for COVID-19 patients, there has been a growing interest on the market place as well as on the research community to develop low cost and real time COVID-19 health monitoring system for Sudanese Ministry of Health. Unfortunately, insufficient rooms in isolation centers and hospitals can cause huge risks on Corona patients. In addition, conventional monitoring techniques cannot provide adequate and accurate monitoring of the patients. Traditionally, health monitoring techniques relied on the direct manual approach to measure vital parameters and monitor patient’s health. However, this manual approach is impractical and costly in terms of time and effort. Moreover, the risk of contracting the disease for the medical staff will increase. Nowadays, Sudanese medical health companies and authorities are looking forward for an in-home Corona health monitoring system that enables good and quick recovery for Corona patients. The present paper seeks to address the performance evaluation for a real time system for monitoring the health status by reading the biomarkers (temperature, blood oxygen saturation, heart rate, and breathing rate) of corona patients remotely and at home using Internet of Things technologies. This work helps and supports COVID-19 patients to monitor their health at home and sending their report according to the system reading remotely with low cost and in real time environment. The obtained experimental data showed that the proposed system is capable of accurately, remotely and reliably monitoring the health monitoring continuously and in a real time with low cost.

## Introduction

Corona Viruses are a group of viruses that can cause diseases such as colds, severe acute respiratory syndrome (SARS) and the Middle East Respiratory Syndrome (MERS) [1–5]. A new type of corona virus has been discovered after being identified as the cause of a widespread disease in China in 2019. This disease is considered one of the most dangerous things in the world, which directly affected many areas and led to high financial losses [6]. The common symptoms of all corona virus strains include fever, dry cough, sore throat and shortness of breath. In fact, healthcare is the process of maintaining or improving health with the help of prevention, diagnosis, treatment for illness and injury. The existing system for healthcare has different limitations which can be summarized as follows. Firstly, patients must go to hospitals for daily health check-ups for knowing regular health status. Secondly, to meet a doctor, the patient has to take the appointment of the doctor days before then he can go for the check-up. Thirdly, this process is not only lengthy but also time consuming. Sometimes even after taking doctor’s appointment, the patient has to wait 2–3 h for meeting the doctors due to which the patient sometimes gets frustrated [7–9]. Finally, most of the conventional healthcare use manual management and maintenance of patient demographic data, case history, diagnostics and medication which leads to human errors and affect patients. Internet of things (IoT) based healthcare overcomes the human errors and helps the physician to diagnose the diseases more easily and accurately by interconnecting all the vital parameters monitoring devices over a network [9–13].

With the increase in the pandemic’s spread and the inability to deal with it in Sudan, isolation centers and hospitals were hitting a breaking point. The current methods used for in-home health monitoring of the patient’s status are not optimal, because of the difficulty in continuous monitoring and supervision [14–17]. The intention is to design and implement a device which can be used for in-home health monitoring for corona virus. In this work, the proposed system consists of four sensors connected together by the use of IoT technology. Two of the sensors will be placed on the patient’s finger and the other two in a mask over patient’s nose and mouth so to measure the vital parameters. The information will then be transferred by the internet and stored over a cloud database which will be analyzed and displayed to the caregivers and the doctors through a graphical user interface (GUI). A threshold of each vital parameter has been set so that an alarming system can call for help immediately. Therefore, real time and remote health monitoring will be provided to a large number of patients.

In fact, Covid-19 has become pandemic, spreading all over the world [18–23]. Scientists and engineers are working day and night to develop a vaccine, to evolve more testing facilities, and to enhance monitoring systems [24–26]. The common symptoms of COVID-19 include fever, dry cough, sore throat and shortness of breath. Thus, health parameters to be monitored are body temperature, blood pressure, respiratory rate and heart pulse.

In this paper, the proposed system was introduced and implemented. The values from the experiment were compared with the reference values of the fingertip pulse oximeter. Moreover, the results obtained from the experiment were presented and discussed. The Wi-Fi module employed in this proposed system was used to transmit data to the “Ubidots” cloud platform which gives the feature of monitoring and logging sensors’ data through the Internet.

Section "Related works" reviews several different popular proposed systems that enable COVID-19 patients to monitor their health at home. In section "Proposed system", the proposed system block diagram, in addition with hardware and software design, is explained in details. Section Results presents the results analysis for the proposed health monitoring system. section "Conclusions" concludes this paper.

## Related works

Researchers have made great efforts to combat Covid-19 by improving the quality of health care to monitor vital parameters and solve the problem of medical staff shortage by using effective techniques that monitor patients remotely to reduce the spread of infection and reduce the burden on hospitals. Various design and implementation procedures have been proposed in order to reach optimal means of monitoring and recording patient status data without the need to frequently visit their doctors with the help of IoT systems.

Smart bracelet proposal has been introduced in [2] to monitor Covid-19 patients in real time based on IoT technologies. They connected the negative temperature coefficient (NTC) body temperature sensor and the pulse oximetry sensor with NodeMCU (open-source firmware and development board based on Wi-Fi module ESP8266-12E). They used the Arduino IDE to develop and analyze data in real time. The wireless network was used to redirect the measurement through a gateway to the cloud. Data are recorded in real time and stored in ThingSpeak Cloud. Global Positioning System (GPS) tracking sensor was used to receive information from satellites and the longitude and latitude coordinates of the bracelet in NMEA format using the Arduino. They used two red and green LEDs on the bracelet to indicate normal and abnormal condition. Arduino sends an alert to the server if a patient's condition worsens.

Similarly, in [3], the authors proposed a technique to detect symptoms of Covid-19 based on the health parameters measurement, which is a measure of the differences in the heartbeats of infected patients. In this system, a wearable sensor is attached to the chest area to monitor and record heart rates. Electrical input was provided through a finger in one hand using a transcutaneous digital electrical nerve simulator. They integrated a GPS module. By using the WIFI module linked to the sensor, the location and data are transmitted in real time to a cloud-based server where they are kept in medical institutions. A heating element connected to the patient's forearm, the temperature level is randomly increased from 32 °C to 50 °C.

In [5], the researchers utilized IoT-based healthcare systems connected through cloud computing and used data analysis to make effective data-driven decision in real time. A single temperature sensor, a NodeMCU board or an Arduino board with sensors and the internet were used. A mobile app was developed using App Inventor which an open-source platform is provided by MIT. ThingSpeak, which is MATLAB's open-source web service API, has been used to store and retrieve data from things using HTTP and MQTT over the Internet or over a local network. The data were read from the sensor in the following sequence: NodeMCU–ThingSpeak–Mobile Applications.

Recently the work done by researchers in [6] introduced a system to detect and monitor COVID-19 patients using Internet of Things devices. The system enables detection of affinity patients by testing blood saturation levels using a pulse oximetry sensor connected to a Raspberry Pi and sending the measurement to clinicians through a mobile phone or laptop. If the level is lower, the blood sample is sent to a PCR test for confirmation. After confirming an infection, the system monitors their condition after isolating them using temperature, blood pressure and heart rate sensors. The collected abnormal values are sent to the doctors through a cell phone or laptop with a GSM module. The values will be displayed on the LCD. The patient details are stored on the web server and from there the alert and detailed information collected through Wi-Fi can be sent to the laptop or desktop.

The pulse oximetry system has been applied in [24]; it is an affordable wireless and remote online monitoring of a Covid-19 patient who was isolated at home. The MAX30100 measuring sensor connected to the RCWL-0530 unit mounted on the patient's finger reads the oxygen saturation and pulse readings and converts the PPG (optical image) signal into a digital signal, which is also converted into numerical values calibrated by the platform processor. The values are sent to the ThingSpeak API data server over the Internet, which broadcasts the received data and collects it using the API service and the identification key. The doctor monitors the patient's health using a smartphone, tablet or PC. ESP32 IoT integrated platform with WIFI capabilities was used. The external communication and sensor control were achieved by the I2C data protocol.

More efforts were done in [25]. Researchers created a system that is a person-wearing bracelet that contains an infrared IR temperature sensor, heart rate sensor, location sensor (GPS), LCD display, Led, battery and Bluetooth module. The information from the sensors is sent via the bracelet to the Arduino board that translates the signals into digital information. This information is not stored on the Arduino Hard Drive because the storage space is insufficient for the large amount of data to monitor the patient. Sensor information is sent to Oracle Cloud.

As a summary of recent work, it is important to find a health monitoring system that provides communication between patient and physician to monitor health status. In fact, there is no single ideal design procedure that can be applied. The basic idea is to design a system to monitor people infected with COVID-19 and monitor their health status without the need for direct contact between them and their doctors. Moreover, many researchers focus on efforts to provide integrated healthcare systems remotely to patients with a level of care and concern that is no less than the level of their presence in the hospital.

## Proposed system

**Fig. 1:**
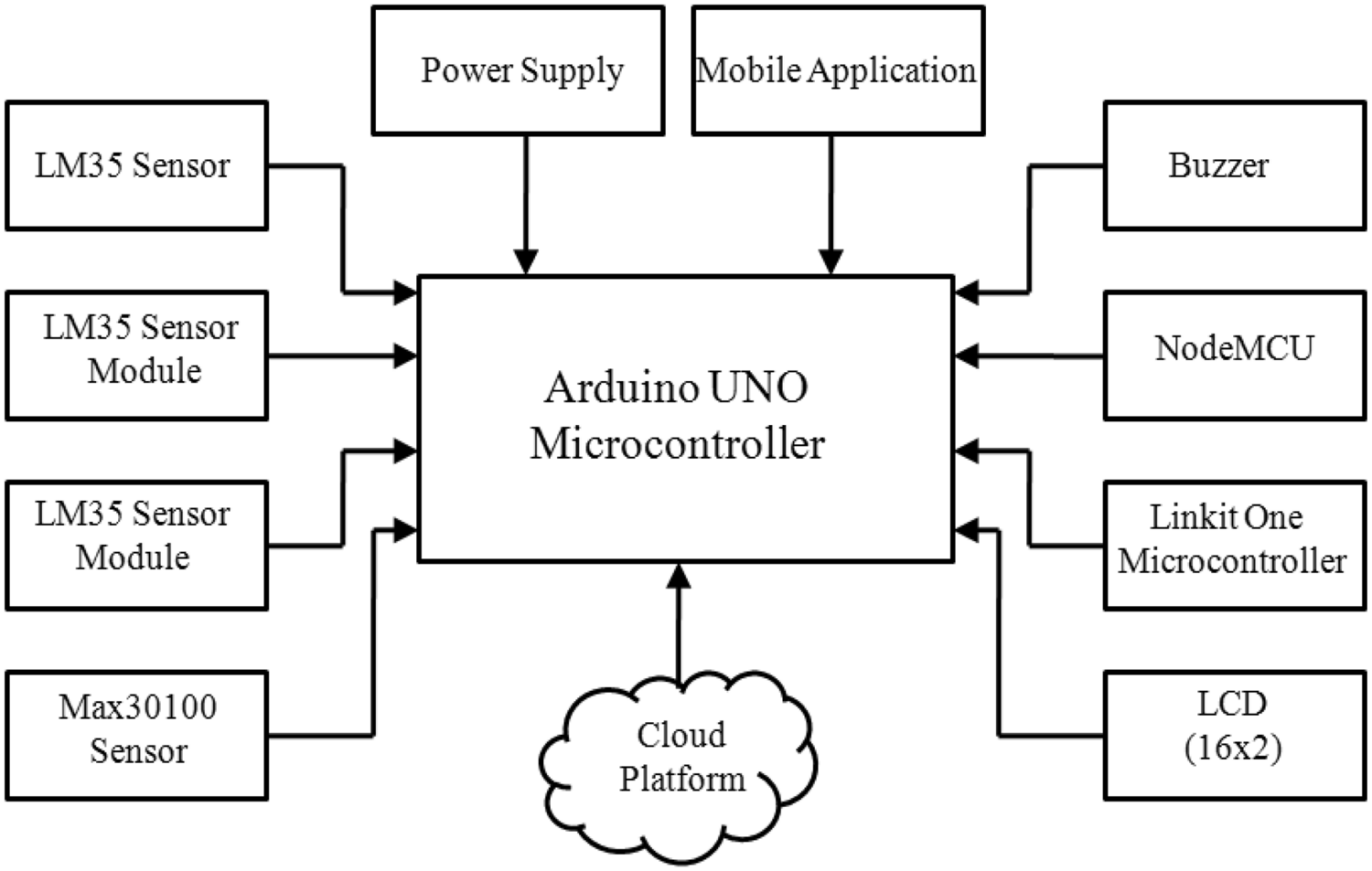
COVID-19 health monitoring proposed system architecture

Various methods have been introduced to present health monitoring system for COVID-19 patients. A design and implementation of real time, online and remote health monitoring system based on IOT technique with low cost have been proposed. The proposed system aims to monitor the health of Corona patient in real time situation and then, notify the risk condition of patient. The remote and real time monitoring/warning capability of the system reduces health risks and the time wasted by the traditional monitoring techniques. In this work as shown in Fig. 1, and to make the system more ubiquitous, it is therefore divided into three subsystems: sensing and perceiving, processing/analyzing and data management/uploading. Additionally, this system is featured with cloud platform to make it more robust and facilitate the monitoring.

### The proposed model functionalities

**Fig. 2:**
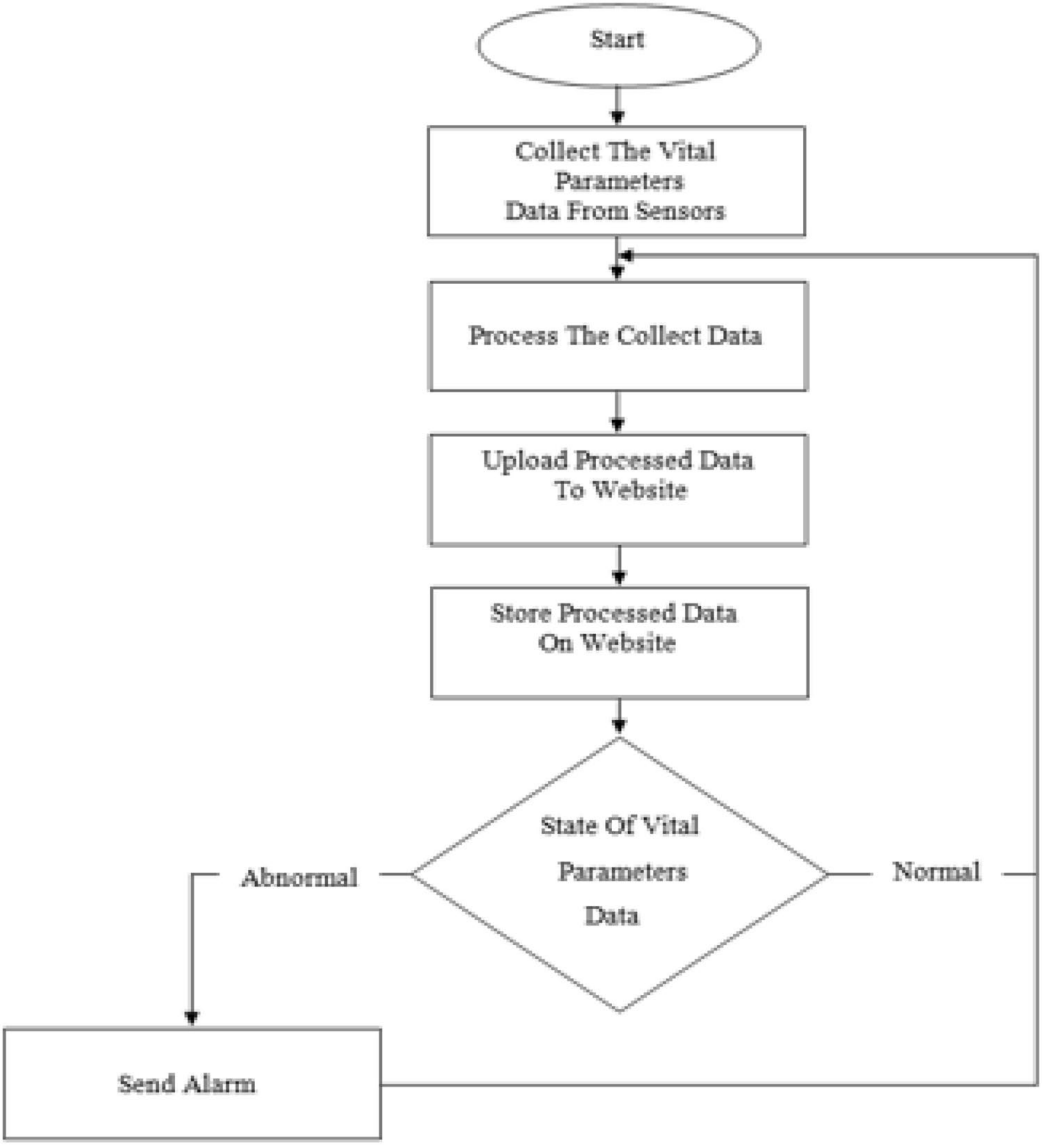
The proposed system flowchart

Various ways have been presented to make a system that takes care of Corona patients in quarantine. Because of the lack of care rooms and the large increase in the number of patients, this work was prepared to be suitable for home quarantine and the doctor's follow-up of his patients from a distance. The proposed method for the remote home health care system is based on sensors, wireless communication technology and IoT cloud platform as presented in Fig. 2.

The first step is the process of collecting data for the vital parameters to be measured and monitored. After a careful survey and according to the WHO guidelines, these general parameters (temperature, heart rate, oxygen percentage in the blood respiration) were decided and deemed appropriate. In fact, the proposed system utilized the following hardware and software components: LinkIt-One microcontroller, Arduino microcontroller, NodeMCU, sensors, LCD screen, buzzer, Arduino IoT cloud platform and Arduino IoT cloud mobile application. The first step is to connect the sensors that read the parameters to microcontroller, which in turn reads and processes the sensors’ data and displays it on the LCD screen, where the sensors are installed on the patient’s finger and respirator mask which is installed over his nose and mouth. In this design, the Arduino microcontroller was chosen because it allows dealing with more than one sensor, whether the output is digital or analog. The second step is the process of sending data to a website in which the data collected in the first step are stored, analyzed and compared to a threshold. If at least one of the values is in an abnormal state, the system will issue an alert after which necessary actions will be taken.

### The proposed subsystems

This section describes in detail the design and implementation of the proposed health surveillance system. Basically, the proposed system can be divided into three subsystems: data collection, data transmission and data management subsystems. Each of these subsystems performs special operations and tasks. In this section, subsystems are explained including the technical concept, main objective and operations performed in each subsystem.

#### Data collection subsystem

This subsystem is considered to collect data. The accuracy of the output (results) mainly depends on its’ performance. It consists of sensors that measure/collect vital parameters, such as temperature (body temperature, breath temperature and room temperature), heart rate and SpO2. Additionally, a patient safety threshold value is assigned. Furthermore, this subsystem includes microcontrollers which come pre-programmed and allows new code to be loaded onto it without the use of an external programmed device and has several tasks that include: converting analog data collected via sensors to digital format using an analog-to-digital converter (ADC), and the values are stored in a temporary memory. It is important to note that these sensors will collect data continuously and present it to the microcontroller periodically according to the frequency of the physiological data of the patient and in turn the controller reads this data and compares it with the pre-set limit values and finally, provides notifications when the parameter value exceeds the threshold limit.

#### Data transmission subsystem

The main objective of this subsystem is to transfer the processed data to the Internet cloud and the mobile application. It mainly connects the other two subsystems through the Wi-Fi module. In fact, Wi-Fi Module is one of the most popular and powerful modules that can be combined with any microcontroller to provide it with internet access, thus enabling it to connect to any cloud platform. Through the use of a Wi-Fi module, a copy of the processed data is sent to a predefined IOT cloud. The uploaded data will be recorded and visualized in the cloud as soon as it arrives.

#### Data management and notification subsystem

This subsystem deals with data management, visualization and notification. The management is implemented remotely, and the values are shown in the form of graphs that provide a visualization of sensor data via real time charts on a web page to serve as a graphical user interface between doctors and from which the doctor will be able to see the condition of patients in real time and can take actions accordingly.

Physiological data and monitoring of vital parameters online can be accessed by the user Using the Internet of Things and stored in the cloud and will be analyzed using software Determine the state of health throughout the day. The doctor is notified by giving an alert by SMS or a recommendation by e-mail if it exceeds the normal condition. Moreover, local and on-site visualization and notification is made possible by this subsystem through two means: an LCD which displays the current values of the relevant quality parameters continuously, a Buzzer for both visual and audible notifications, respectively, whenever a parameter value exceeds the threshold. However, the user can choose whether to activate or deactivate the audible alarm through enabling the mute mode by pressing an embedded button. After that Signals are sent to find a person's direct location on GPS.

## Results

In this section, an experiment was carried out to evaluate the proposed system performance. The setup and principal findings of this experiment are discussed in the following subsections.

### Practical experience

**Fig. 3:**
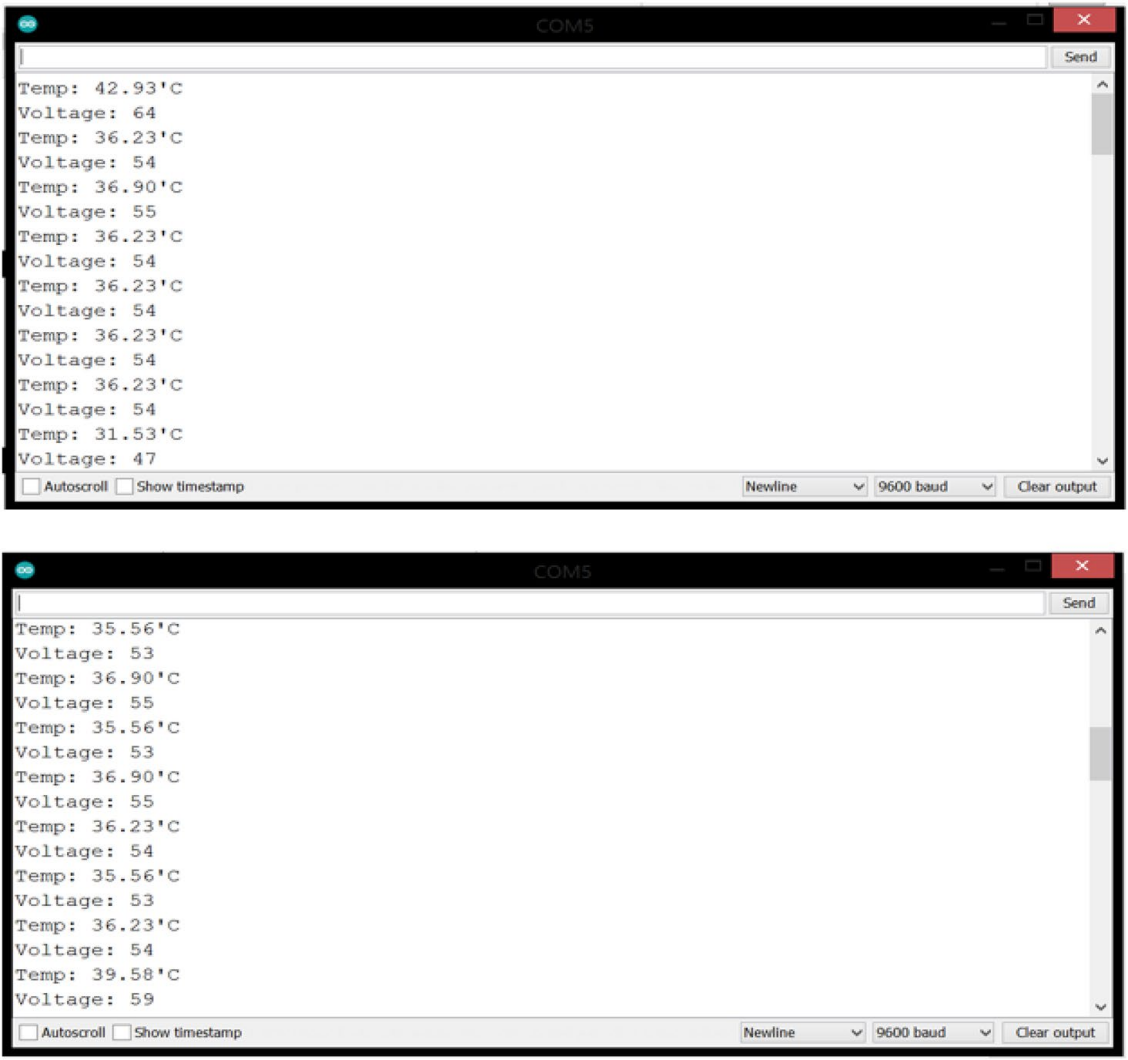
LM35 reading

**Fig. 4:**
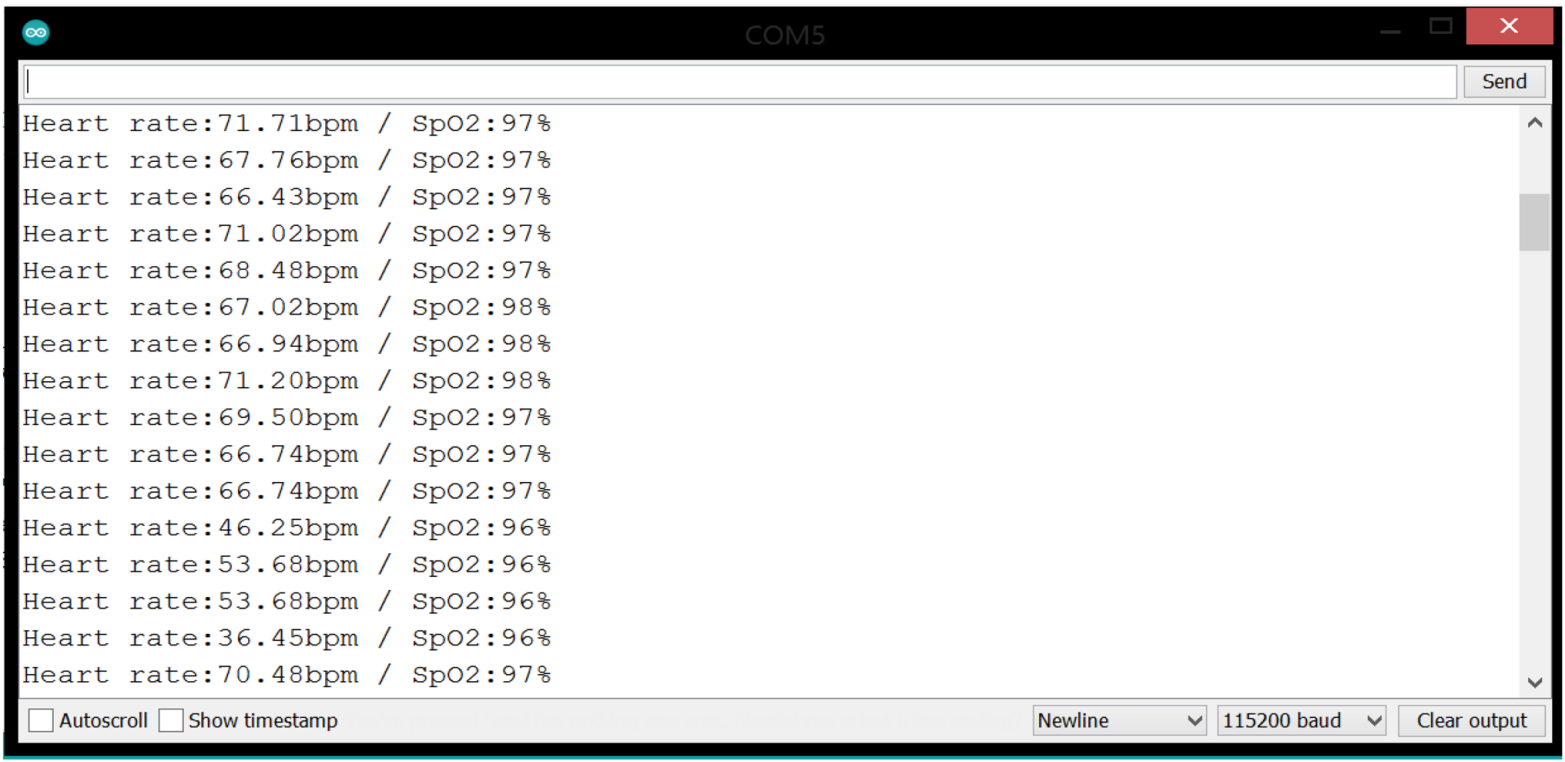
MAX30100 reading

After the system circuit was connected, it was fed with a power source, which is the laptop via an USB cable. A plastic wrap containing the LM35 and MAX30100 sensors has been installed in the patient’s finger, and the respiratory mask is placed so that it covers the mouth and nose. One of the LM35 sensors was placed outside of the mask, and another one was installed inside the mask. Due to the unavailability of a patient infected with covid-19, the readings of temperature, heart rate and blood oxygen percentage were taken from a normal, uninfected person as shown in Figs. 3 and 4.

**Fig. 5:**
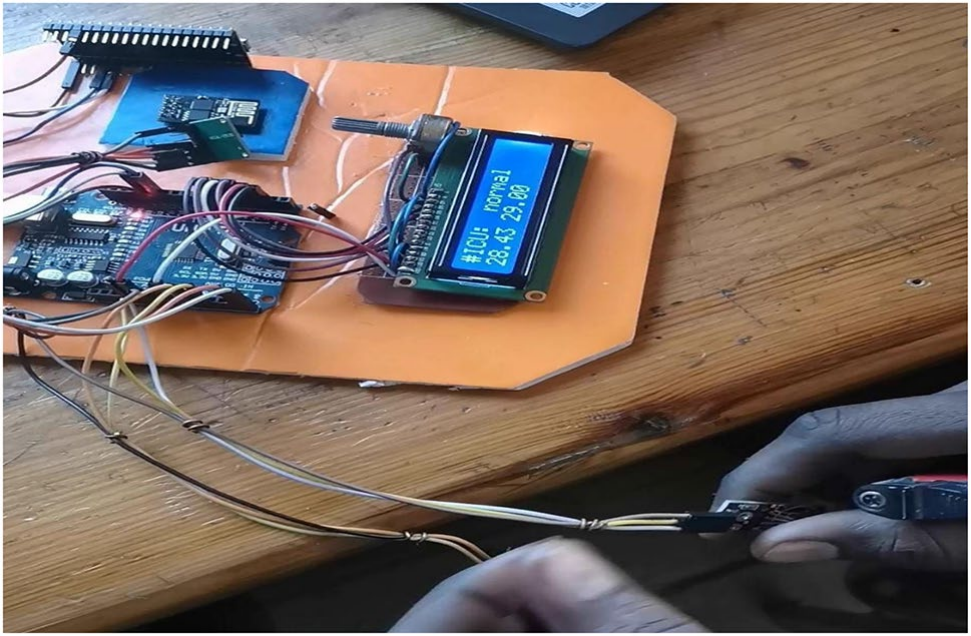
Breathing circuit in a normal condition

The process of respiration consists of inhaling oxygen through inhalation and exhaling carbon dioxide through exhalation. It is worth noting that the exhaled temperature is the same as the body temperature, as it was found that during normal breathing, the difference between the temperature of the carbon dioxide released during the breathing process and the surrounding atmosphere is 10 degrees, and if the difference is disturbed, breathing is considered abnormal. The proposed breathing circuit determines whether breathing is normal or not, as it works by taking the LM35 temperature sensor installed on the outside of the mask for room temperature and subtracting it from the breathing temperature measured by the LM35 temperature sensor installed inside the mask. As it was mentioned earlier that no patient with covid-19 was provided, so the breathing circuit using a lighter was tried. As shown in Fig. 5, when the lighter is removed from the sensor, the temperature difference is 10 degrees, and the normal condition is shown on the screen.

**Fig. 6:**
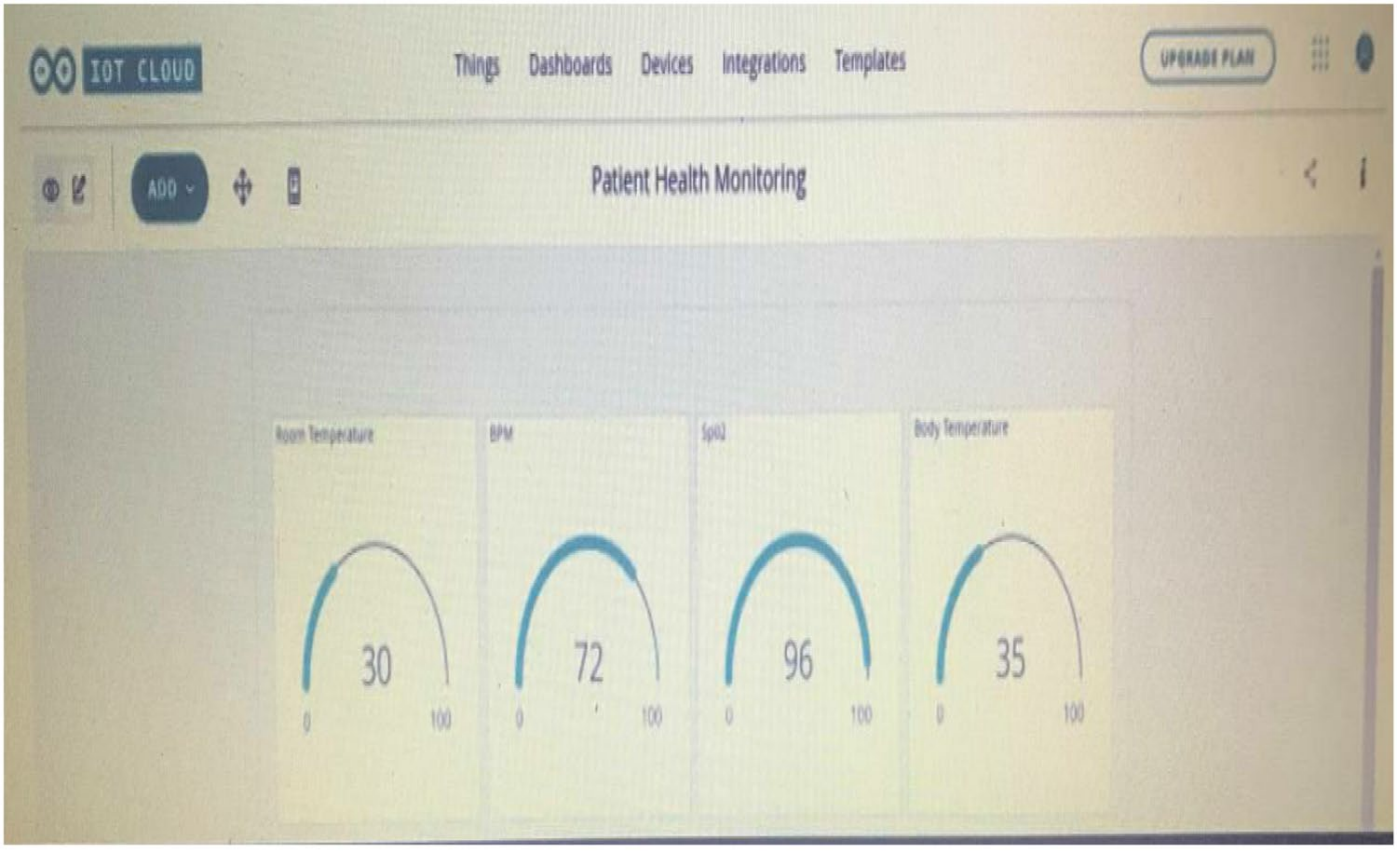
Arduino IoT platform

After the vital parameter data are taken and uploaded to the Arduino IoT platform via the NodeMCU, it is displayed in real time as shown in Fig. 6.

In the event that at least one of the vital parameters is in an abnormal state, the system alarms by means of a buzzer and it causes pin A9 to be activated to 1 in Arduino. Then, automatically, pin A13 is activated in the LinkIt one, which sends an alert text message.

#### Proposed system evaluation

The main objective of this section is to evaluate the performance of the proposed system, where the fingertip pulse oximeter was used as an evaluation criterion and when tested it was found that the average values of the vital parameters taken was 35.6° C for body temperature, 97% for SPO2 and 70 BPM for heart rate.

Through the experiment that was conducted and the results obtained from this proposed system, it was found that the values of the parameters are close to those taken from the fingertip pulse oximeter. The results are shown in Table 1. Heart rate is measured at a rate of beats per minute (BPM), so the sudden drop that occurred in the readings of this vital parameter is due to the fact that these results are taken with reporting period of 1 s.

**Table 1:** Vital parameters results

Temperature (°C)	SPO2 (%)	Heart rate (BPM)
42.93	95	67.86
36.23	95	64.21
36.9	95	64.21
36.23	97	42.05
36.23	97	38.98
36.23	97	38.98
31.53	97	38.98
35.56	97	51.88
36.9	97	51.88
36.56	97	36.13
36.9	97	47.05
36.23	97	63.97
35.56	97	67.92
39.58	97	67.3
33.54	97	67.01
36.23	97	69.35
36.23	97	71.71
35.56	97	67.76
36.23	97	66.43
36.23	97	71.02

## Conclusions

The main goal of the present paper was to experimentally evaluate the performance of a low-cost real time COVID-19 health monitoring system for Sudanese ministry of health. In the traditional system, health monitoring of patients infected with this dangerous virus widely spread all over the world requires a manual approach by the medical staff, but this method puts more life in the face of disease risks as well as errors in data collection and preservation. Moreover, this method is time consuming and labor intensive. Therefore, there is an urgent need to monitor COVID-19 patients in real time in order to monitor their health care in an appropriate setting. At present, the World Health Organization Medical Research Center is very interested in finding a remote home healthcare system for COVID-19. Telehealth facilities have become a frontline force in the struggle to reduce the spread of healthcare-associated COVID-19 and ultimately protect healthcare professionals. While telehealth can replace efficiency and accessibility, its reliance on real time communication of medical records via the cloud. So, a low-cost and real time COVID-19 health monitoring system has been suggested through the Internet and depends on the technology of the Internet of Things. The proposed system aims to monitor the health of corona patients at home and in real time and notify the doctor when at least one of the vital parameters becomes abnormal. In this work, four different vital parameters were monitored: temperature, blood oxygen content (SPO2), heart rate and respiration. The system's ability to remote, automatic and real time monitoring/alert reduces health risks. The proposed system consists of three subsystems: data collection, transmission and management subsystems. The main idea is to install a set of sensors on the patient's body that will monitor his vital parameters. The system contains a proposal for the possibility of remote monitoring and directing the patient or his caregiver to the correct decisions, or the provision of a specialized doctor.

## Data Availability

The datasets generated during and/or analyzed during the current study are available from the corresponding author on reasonable request.

## Availability of code

The codes generated during the current study are available from the corresponding author on reasonable request.

## References

[1] Jaafari S, Alhasani A, Almutairi SM (2020) Certain investigations on IoT system for COVID-19. In: 2020 International conference on computing and information technology (ICCIT-1441), IEEE, pp 1–4

[2] Acharya AD, Patil SN (2020) IoT-based health care monitoring kit. In: 2020 Fourth international conference on computing methodologies and communication (ICCMC), IEEE, pp 363–368

[3] Vishnu S, Ramson SJ, Jegan R (2020) Internet of medical things (IoMT): an overview. In: 2020 5th international conference on devices, circuits and systems (ICDCS), IEEE, pp 101–104

[4] AlShorman O, AlShorman B, Alkhassaweneh M, Alkahtani F (2020). A review of internet of medical things (IoMT)–based remote health monitoring through wearable sensors: a case study for diabetic patients. Indonesian J Electr Eng Comput Sci.

[5] Siam AI, Abou Elazm A, El-Bahnasawy NA, El Banby G, Abd El-Samie FE, Abd El-Samie F (2019) Smart health monitoring system based on IoT and cloud computing. Menoufia J Electron Eng Res 28(1):37–42

[6] Wang W-Y, Xie Y, Zhou H, Liu L (2020) Contribution of traditional Chinese medicine to the treatment of COVID-19. Phytomedicine, 153279 10.1016/j.phymed.2020.153279 PMC7338274 32675044

[7] Kumar K, Kumar N, Shah R (2020). Role of IoT to avoid spreading of COVID-19. Int J Intell Netw.

[8] Schofield J, Leelarathna L, Thabit H (2020). COVID-19: impact of and on diabetes. Diabetes Therapy.

[9] Ullah W, Yahya A, Samikannu R, Tlale T (2021) Robust and secured telehealth system for COVID-19 patients. In: Data Science for COVID-19, Elsevier, pp 337–349

[10] Vangeti M, Wata Dereso C, Rudrapati R, Akhila M, Muturaman M (2020) Applications of internet of things (IoT) to track COVID-19 in real time. Int J Adv Res Eng Technol 11(9)

[11] Yogesh D, Periasamy A, Karuppiah T. IoT based wing commander pilot emergency live tracking and ECG monitoring wrist band

[12] Abusaada H, Elshater A (2020). COVID-19 challenge, information technologies, and smart cities: considerations for well-being. Int J Commun Well-being.

[13] Ennafiri M, Mazri T (2020) Internet of things for smart healthcare: a review on a potential IOT based system and technologies to control COVID-19 pandemic. In: The proceedings of the third international conference on smart city applications, Springer, pp 1256–1269

[14] Josephine M, Lakshmanan L, Nair RR, Visu P, Ganesan R, Jothikumar R (2020) Monitoring and sensing COVID-19 symptoms as a precaution using electronic wearable devices. Int J Pervasive Comput Commun

[15] Arun M, Baraneetharan E, Kanchana A, Prabu S (2020) Detection and monitoring of the asymptotic COVID-19 patients using IoT devices and sensors. Int J Pervasive Comput Commun

[16] Sahandi R, Noroozi S, Roushan G, Heaslip V, Liu Y (2010). Wireless technology in the evolution of patient monitoring on general hospital wards. J Med Eng Technol.

[17] Gupta D, Bhatt S, Gupta M, Tosun AS (2021). Future smart connected communities to fight covid-19 outbreak. Internet Things.

[18] Zhu H (2019). Smart healthcare in the era of internet-of-things. IEEE Consumer Electron Magazine.

[19] ENISA (2016) Smart hospitals: security and resilience for smart health service and infrastructures. ed: ENISA Heraklion, Greece

[20] Moro Visconti R, Martiniello L (2019) Smart hospitals and patient-centered governance. In: Moro Visconti R, Martiniello L (2019) Smart hospitals and patient-centered governance. Corporate Ownership & Control, 16(2)

[21] Vianello A (2013). “Hospital at home” for neuromuscular disease patients with respiratory tract infection: a pilot study. Respir Care.

[22] Leff B (2006). Satisfaction with hospital at home care. J Am Geriatr Soc.

[23] Malasinghe LP, Ramzan N, Dahal K (2019). Remote patient monitoring: a comprehensive study. J Ambient Intell Humaniz Comput.

[24] Miron-alexe V (2020). IoT pulse oximetry status monitoring for home quarantined COVID-19 patients. J Sci Arts.

[25] Cacovean D, Ioana I, Nitulescu G (2020). IoT system in diagnosis of Covid-19 patients. Inform Econ.

[26] Taiwo O, Ezugwu AE (2020). Smart healthcare support for remote patient monitoring during covid-19 quarantine. Inform Med Unlocked.

